# Pharmacological Targeting of Native CatSper Channels Reveals a Required Role in Maintenance of Sperm Hyperactivation

**DOI:** 10.1371/journal.pone.0006844

**Published:** 2009-08-31

**Authors:** Anne E. Carlson, Lindsey A. Burnett, Donato del Camino, Timothy A. Quill, Bertil Hille, Jayhong A. Chong, Magdalene M. Moran, Donner F. Babcock

**Affiliations:** 1 Department of Physiology and Biophysics, University of Washington, Seattle, Washington, United States of America; 2 Hydra Biosciences Inc., Cambridge, Massachusetts, United States of America; 3 Cecil H. and Ida Green Center for Reproductive Biology Sciences, Department of Pharmacology, University of Texas Southwestern Medical Center, Dallas, Texas, United States of America; Monash University, Australia

## Abstract

The four sperm-specific CatSper ion channel proteins are required for hyperactivated motility and male fertility, and for Ca^2+^ entry evoked by alkaline depolarization. In the absence of external Ca^2+^, Na^+^ carries current through CatSper channels in voltage-clamped sperm. Here we show that CatSper channel activity can be monitored optically with the [Na^+^]_i_-reporting probe SBFI in populations of intact sperm. Removal of external Ca^2+^ increases SBFI signals in wild-type but not CatSper2-null sperm. The rate of the indicated rise of [Na^+^]_i_ is greater for sperm alkalinized with NH_4_Cl than for sperm acidified with propionic acid, reflecting the alkaline-promoted signature property of CatSper currents. In contrast, the [Na^+^]_i_ rise is slowed by candidate CatSper blocker HC-056456 (IC_50_ ∼3 µM). HC-056456 similarly slows the rise of [Ca^2+^]_i_ that is evoked by alkaline depolarization and reported by fura-2. HC-056456 also selectively and reversibly decreased CatSper currents recorded from patch-clamped sperm. HC-056456 does not prevent activation of motility by HCO_3_^−^ but does prevent the development of hyperactivated motility by capacitating incubations, thus producing a phenocopy of the CatSper-null sperm. When applied to hyperactivated sperm, HC-056456 causes a rapid, reversible loss of flagellar waveform asymmetry, similar to the loss that occurs when Ca^2+^ entry through the CatSper channel is terminated by removal of external Ca^2+^. Thus, open CatSper channels and entry of external Ca^2+^ through them sustains hyperactivated motility. These results indicate that pharmacological targeting of the CatSper channel may impose a selective late-stage block to fertility, and that high-throughput screening with an optical reporter of CatSper channel activity may identify additional selective blockers with potential for male-directed contraception.

## Introduction

We have known for many years that Ca^2+^ and cAMP messengers play central roles in the preparation of sperm for fertilization in the physiologic sequence that is collectively called ‘capacitation’ [1]. However, the last 5 years have brought a great increase in our knowledge of the details of Ca^2+^ and cAMP signaling in sperm. The advances resulted largely from discovery that signaling in sperm uses several unique proteins that are produced primarily or exclusively in meiotic and post-meiotic spermatogenic cells, opening the door for genetic approaches to study sperm signaling pathways and their roles. Thus, targeted disruption of the genes for the sperm-specific Cα2 catalytic subunit of PKA [2] or for SACY, the atypical adenylyl cyclase of sperm [3]–[5], produced male infertility. The specific phenotypic defects that were found in the null-mutant sperm revealed a dichotomy: acceleration of the flagellar beat and facilitation of evoked Ca^2+^ channel activity required SACY [5], [6] and PKA-Cα2 [2], whereas a basal flagellar beat and a basal rate of evoked Ca^2+^ channel activity did not.

Similar advances in our understanding of Ca^2+^ signaling in sperm followed discovery that spermatogenic cells express a novel putative Ca^2+^ channel formed by the four members of the unique family of CatSper ion channel proteins. CatSper family proteins are expressed exclusively in spermatogenic cells and specifically traffic to membranes of the developing sperm flagellum. Targeted disruption of CatSper1 [7], CatSper2 [8], or of CatSper3 or CatSper4 [9], [10] each produced male infertility. The seemingly identical phenotypes of the CatSper family null-mutant sperm result from the co-dependent expression of the CatSper-1 and −2 [10], [11] and of the CatSper-3 and −4 [10] proteins. A physical association of these proteins with each other and with other interacting partners [12] presumably allows them to form functional channels and may stabilize them from degradation. Like the SACY-null sperm, CatSper-null sperm initiate and sustain a basal flagellar beat [11], [13]. However, unlike SACY-null sperm, the HCO_3_^−^ anion still accelerates the flagellar beat of CatSper1- and −2 null sperm indicating that downstream cAMP-mediated responses are unperturbed by the absence of CatSper proteins. Conversely, the CatSper-null sperm lack depolarization-evoked Ca^2+^ entry [11], [14] and the inward Ca^2+^ current that is observed in wild-type sperm [7], [9], [15]. They also fail to develop hyperactivated motility [8]–[11], [14]. These results indicated that CatSper channels are required as the route for the Ca^2+^ entry needed to produce the asymmetric flagellar waveform that underlies hyperactivation.

Although the loss-of-function phenotype provides definitive evidence of a required role for CatSper channels in hyperactivation, it has remained unclear whether the requirement is for the onset of hyperactivation, for its maintenance, or for both. We have now identified HC-056456 as a blocker of CatSper channel activity and find no hyperactivation for sperm that receive capacitating incubations in its presence. In this and other respects, HC-056456 produces a pharmacological phenocopy of the CatSper-null sperm. We also find that acute application of HC-056456 causes rapid loss of flagellar waveform asymmetry from hyperactivated sperm, indicating that continued entry of Ca^2+^ through CatSper channels is required to maintain hyperactivation.

Although monitoring of CatSper currents in patch-clamped sperm is the definitive method to establish channel-blocking activity, an inability to express CatSper channels in cultured cells has prevented large-scale screening of chemical libraries for Catsper channel blockers. We have now validated an optical method that reports activity of native CatSper channels in populations of intact sperm. The method should be readily adaptable to high-throughput screening protocols.

## Materials and Methods

### Chemicals

Fura-2 acetoxymethyl (AM) ester, SBFI-AM, and Pluronic F127 were from Molecular Probes (Eugene, OR). HC-056456 (3,4-bis(2-thienylcarbonyl)-1,2,5-oxadiazole-2-ium-2-olate; for structure, see Supplemental Fig. S1) was from Maybridge Chemical Co. Ltd. (Tintagel, Cornwall, UK). All other chemicals were from Sigma (St. Louis, MO).

### Animals and Cell Preparation

All animal procedures followed accepted standards of humane animal care, approved by the Animal Care and Use Committee at the University of Washington. As in prior work [16], [17], sperm were collected from excised caudae epididymides and vasa deferentia of euthanized male mice. Sperm were washed twice then dispersed and stored at 1−2×10^7^ cells ml^−1^ at room temperature (22–25°C) in medium HS [11], [17]. Some experiments used modified HS medium with no added CaCl_2_ (‘0 mM Ca^2+^’ treatments). Other experiments used a sodium-free medium that contained (in mM): 135 N-methyl-D-glucamine (NMDG), 5 KCl, 2 CaCl_2_, 1 MgSO_4_, 10 glucose, 20 HEPES, adjusted pH to 7.4 with KOH. As in past work [14], [18], depolarization-evoked responses were produced with alkaline medium K8.6 and hyperactivation was produced by 90 min of incubation under capacitating conditions. Capacitated sperm were then transferred back into medium HS and sampled for waveform analysis within 30–45 min.

All waveform analyses and photometric experiments were conducted in glass-bottomed 35 mm culture dishes. For local perfusion experiments, test solutions were applied by a solenoid-controlled, gravity-fed, multi-barreled local perfusion device with an estimated exchange time of <0.5 s.

### Waveform Analysis

The flagellar waveform was analyzed as described [17]. Briefly, stop-motion images were captured with stroboscopic (1 ms) illumination. A semi-automated analysis program written in Igor Pro (Wavemetrics, Lake Oswego, OR) allowed tracing of the flagellum and determination of various waveform parameters including flagellar beat frequency and asymmetry. Here we introduce the asymmetry integral (derived by integration of the time-averaged tangent angle over the proximal half of the flagellum), as a more-inclusive measure of the bias of flagellar bending. All results are presented as mean±SEM, except where noted.

### Dye Loading and Calcium and Sodium Photometry

Fura-2-AM and SBFI-AM were dispensed from 2 mM stocks in DMSO, dispersed in 10–15% Pluronic F127, and diluted to 20 µM in 0.5 ml of sperm suspension. After 30–40 min, cells were sedimented, then resuspended and incubated in fresh HS for an additional 45 min before use. A TILL (Gräfelfing, Germany) monochromator provided excitation light of 340 and 380 nm, and a photodiode detector with an adjustable viewfinder collected >450 nm emission from a rectangular region containing a small cluster (3–5 cells) of loosely-tethered sperm. The raw F_340_ and F_380_ photometric signals were corrected for the cell-free background, collected prior to each series of measurements.

The ratio of the corrected fura-2 signals, R_fura-2_, was calibrated as described previously using the constants: B = 1228 nM, R_min_ = 0.3795, and R_max_ = 1.792 [11], [14]. The calibrated R_fura2_ signal reports spatially-averaged internal [Ca^2+^] from the head and proximal flagellum of several sperm. The background-corrected but uncalibrated R_SBFI_ (F_340_/F_380_) signals report relative internal [Na^+^]. Statistical analyses were performed in Excel (Microsoft, Redmond, WA).

### Electrophysiological recording of CatSper and KSper currents

Mouse sperm were isolated from freshly dissected mouse epididymides and incubated in buffer HS. Macroscopic currents were recorded in the whole sperm configuration of the patch-clamp technique using an EPC-9 amplifier and Patchmaster software (HEKA). Patch pipettes had a resistance of 7–14 MΩ.

For CatSper currents recording conditions were essentially as described in Kirichok, et al. [15]. The pipette solution contained (in mM): 140 Cs-MES, 5 NaCl, 10 EGTA, 10 HEPES pH 7.2 with NaOH. The bath contained (in mM) 150 Na-Gluconate, 2 EGTA, 2 H-EDTA, 20 HEPES, pH 7.4 with NaOH. KSper currents were recorded using methods similar to those described in Navarro et al. [19]. Pipettes were filled with an internal solution (in mM): 5 EGTA, 10 HEPES, 10 MES, 140 K-MeSO_3_, 10 MES, pH 8 with KOH. The bath solution contained (in mM): 150 K- MeSO_3_, 10 MES, 10 HEPES, pH 7.2 with KOH. The holding potential was 0 mV, and 400-ms ramps from −100 mV to 100 mV were applied every 5 s. The data were collected at 10 kHz and filtered at 2.9 kHz. Liquid junction potential correction was not used. Experiments were performed at room temperature. HC-056456 was prepared fresh as a 10–50 mM stock solutions in DMSO and diluted into the external buffer immediately before application to the sperm.

## Results

### Removal of external Ca^2+^ allows intracellular Na^+^ to rise

**Figure 1:**
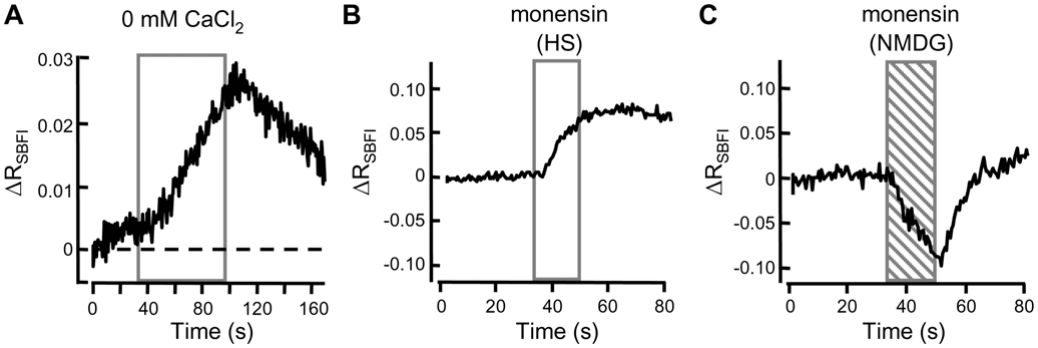
Intracellular Na^+^ increases evoked by removal of external Ca^2+^. Averaged 340/380 fluorescence ratio signals from the [Na^+^]_i_ probe SBFI. The initial ratio (R_0_ = 0.59–0.66; observed in the first 10 s of each recording) was subtracted from all R_SBFI_ values. The normalized ΔR_SBFI_ data were then averaged. (A) Sperm were perfused with medium HS, then for 60 s (boxed) with Ca^2+^-deficient HS (79 clusters of cells in 5 independent experiments). (B,C) Sperm were perfused with medium HS, then for 30 s (boxed) with medium HS or with NMDG-substituted (Na^+^-deficient) HS medium each fortified with 10 µM monensin (8–25 clusters of cells in 2 independent experiments).

In past work, we found that the CatSper channel of sperm is the predominant route for the entry of Ca^2+^ into sperm that is evoked by alkaline depolarizing medium K8.6 [11], [14]. We wanted to develop a simple optical assay for CatSper activity. Ca^2+^ channels, including CatSper [9], [15], readily pass Na^+^ in the absence of their preferred Ca^2+^ ion. We therefore reasoned that we could optically monitor Na^+^-entry via the CatSper channel into intact sperm examined in Ca^2+^-free solutions. For sperm loaded with the [Na^+^]_i_ probe SBFI (Fig. 1A), the ratiometric SBFI signal (R_SBFI_) remained constant while cells were bathed with medium HS, whereas the R_SBFI_ signal increased at a rate of ∼0.02 ΔR_SBFI_ min^−1^ upon acute exposure to a Ca^2+^-deficient HS. The Na^+^ entry indicated by the rising R_SBFI_ stopped abruptly when Ca^2+^ was returned and [Na^+^]_i_ began to return to the resting [Na^+^]_i_.

The [Na^+^]_i_ sensitivity of the R_SBFI_ signal was validated by use of the Na^+^-selective ionophore monensin. The R_SBFI_ increased in response to the Na^+^ entry that occurred upon addition of monensin to cells in medium HS (Fig. 1B) and decreased when monensin promoted Na^+^ release into a Na^+^-deficient (NMDG-substituted) medium (Fig. 1C). When monensin and NMDG^+^ were removed from the perfusing medium, [Na^+^]_i_ rose rapidly, driven by the restored inward-directed [Na^+^] gradient and mediated by monensin retained in sperm membranes. Removal of monensin from the HS medium used in Fig. 1B initiated a much slower recovery towards the initial [Na^+^]_i,_. Presumably, continued entry of Na^+^ mediated by residual membrane-associated monensin continued to oppose cellular mechanisms for extrusion of Na^+^ and restoration of resting [Na^+^]_i_. Visual observation indicated that the sperm flagellar beat was slowed but still present after monensin treatment, thus membrane integrity and cellular SBFI content had not been compromised.

Although SBFI is widely used to monitor [Na^+^]_i_, the dye is notoriously difficult to calibrate (see Meier et al., 2006 [20]). In Fig. 1B the R_SBFI_ increased by ∼0.06 when monensin discharged transmembrane gradients of [Na^+^] and internal [Na^+^] rose to the >100 mM contained in the HS medium. In Fig. 1C the R_SBFI_ decreased by ∼0.08 when monensin lowered internal [Na^+^] towards the <1 mM Na^+^ present in the NMDG medium. These results are consistent with an initial resting internal [Na^+^] slightly above the K_d_ of ∼10 mM Na^+^ for SBFI in vitro (and close to the 14 mM [Na^+^]_i_ reported for sperm using an alternative optical method [21]). Based on these approximations, we estimate that internal [Na^+^] rose to 40–50 mM during the 60 s zero-calcium stimulus applied in Fig. 1A.

### Na^+^ entry requires CatSper

**Figure 2:**
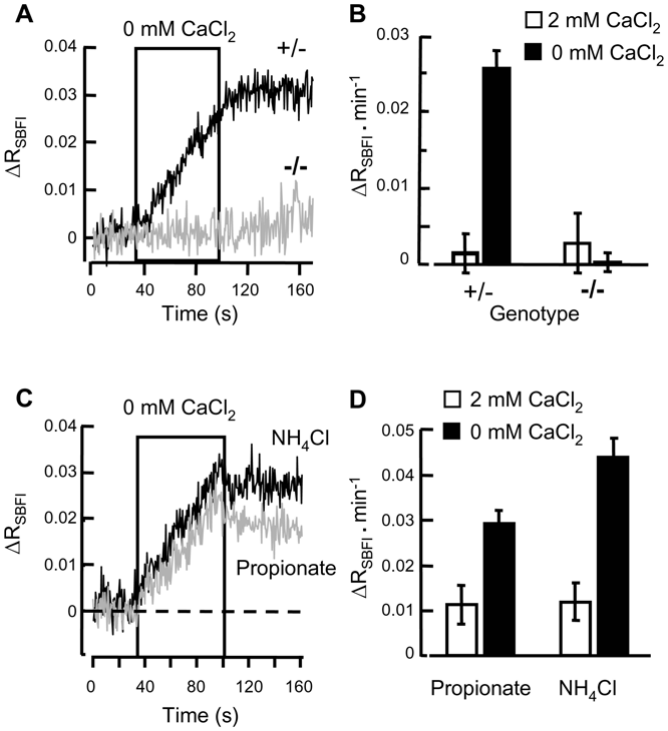
Evoked [Na^+^]_i_ rise requires CatSper2. Responses of the SBFI probe monitored from small clusters of CatSper2-null sperm (gray trace, −/−) and of sperm from their heterozygous littermates (black trace, +/−). (A) Cells were perfused first with medium HS, then for 60 s (boxed) with Ca^2+^-deficient HS. Lines are averages of normalized ΔR_SBFI_ data. (B) Averaged rate of change in ΔR_SBFI_ before and during perfusion with Ca^2+^-deficient HS medium (49–50 cell clusters in 3 independent experiments). (C) Wild-type sperm were perfused with medium HS then for 60 s (boxed) with Ca^2+^-deficient HS, both fortified with 15 mM NH_4_Cl (black traces) or 15 mM Na propionate (gray traces). (D) Averaged normalized rates of rise of ΔR_SBFI_ (9–14 groups of cells in 3 independent experiments).

To more directly test the hypothesis that the rise in [Na^+^]_i_ results from entry of Na^+^ via the CatSper channel, we compared the SBFI responses in sperm from CatSper2^−/−^ males with those from their CatSper2^+/−^ littermates. Upon removal of external Ca^2+^, the R_SBFI_ in CatSper2^−/−^ sperm remained constant, but it increased at 0.03 ΔR_SBFI_ min^−1^ in CatSper2^+/−^ sperm (Fig. 2A,B). CatSper2^−/−^ sperm lack both CatSper1 and CatSper2 proteins, yet they retain normal immunolocalization of Ca_V_ channels [11]. Therefore, the loss of entry of Na^+^ evoked by removal of external Ca^2+^ results specifically from the absence of CatSper channel proteins.

### Raising internal pH promotes Na^+^ entry

We sought additional evidence that the increase in [Na^+^]_i_ evoked by Ca^2+^-free solution (Figs. 2A,B) shares characteristics with CatSper currents. CatSper current is responsive to changes in intracellular pH; intracellular acidification reduced, and alkalinization increased, the CatSper-dependent Na^+^ currents recorded from voltage-clamped sperm [15]. Figs. 2C,D show that for intact sperm monitored with SBFI and challenged with Ca^2+^-deficient medium, the rate of rise (ΔR_SBFI_) was 0.045±0.005 min^−1^ in ammonium-supplemented medium and 0.030±0.002 min^−1^ in proprionate-supplemented medium. Such brief treatments with 15 mM NH_4_Cl increased spatially-averaged sperm pH_i_ from ∼6.8 to ∼7.2, whereas treatments with propionate decreased pH_i_ to ∼6.4 [16]. Thus the rise in [Na^+^]_i_ evoked in intact sperm shows the property of promotion by alkalinization that is a signature of CatSper currents in patch-clamped sperm [15].

### HC-056456 blocks evoked Ca^2+^and Na^+^ entry

**Figure 3:**
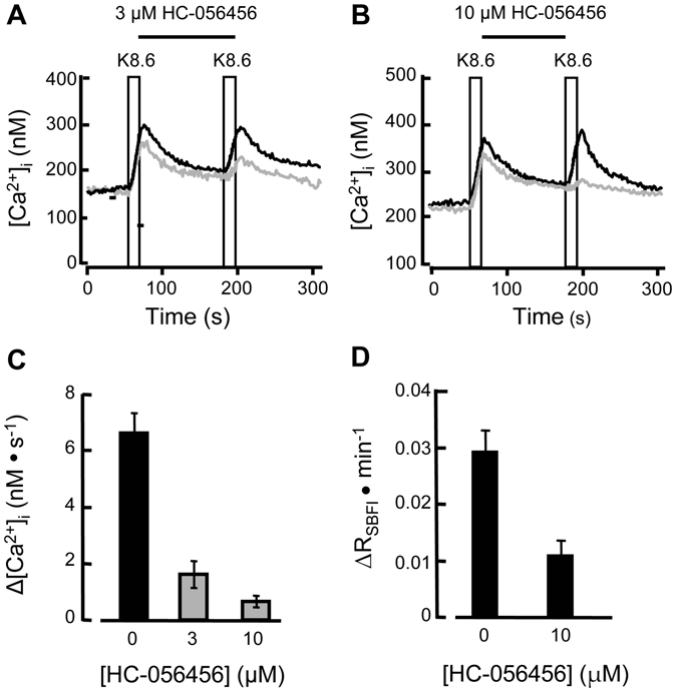
HC-056456 blocks alkaline KCl-evoked increases in [Ca^2+^]_i_ and [Na^+^]_i_. (A,B) Intracellular [Ca^2+^] for sperm perfused with HS alone (black traces) except during two 10 s stimuli with medium K8.6 (boxed). In a similar protocol using fresh sperm from the same animal, the inter-pulse medium HS also contained 3 or 10 µM of HC-056456 (gray traces). In (A) the HC-056456 also was present in the K8.6 medium used for the second stimulus. (16–19 groups of cells in 3 independent experiments). (C) Averaged rates of rise for [Ca^2+^]_i_ during the second stimulus. (D) Intracellular [Na^+^]_i_ was monitored for cell clusters before and during a 60 s stimulus with Ca^2+^-deficient HS medium as in Fig. 2. Parallel experiments were conducted with or without 10 µM HC-056456 present in all perfusing media. Averaged rate of change ΔR_SBFI_ was calculated as for Fig. 2 (13–14 groups of cells in 3 independent experiments).

The CatSper channel is the predominant route for evoked Ca^2+^ entry into intact mouse sperm [11], [14]. Compound HC-056456 was identified as a potential inhibitor of CatSper in a chemical-library screen (see United States Patent applications 20070004785 and 20080311578). We therefore examined the efficacy of HC-056456 to inhibit evoked Ca^2+^ entry. In Figs. 3A–C, fura-2 reported the spatially-averaged [Ca^2+^]_i_ of sperm during challenge with a paired-stimulus protocol. Medium K8.6 evoked averaged rates of rise of 8.7 and 6.6 nM Δ[Ca^2+^]_i_ s^−1^ during the first and second stimuli when no inhibitor was present. When 3 µM HC-056456 was applied after the first stimulus, the averaged rate decreased from ∼7.5 to ∼1.6 nM Δ[Ca^2+^]_i_ s^−1^. When 10 µM HC-056456 was applied after the first stimulus, the averaged rate decreased more strongly, from ∼7.0 to ∼0.7 nM Δ[Ca^2+^]_i_ s^−1^. Hence HC-056456 is an effective inhibitor of the CatSper Ca^2+^ channel activity that is evoked by alkaline depolarization. Fig. 3D shows that at 10 µM the HC-056456 also strongly decreased the rise in [Na^+^]_i_ that was evoked by removal of external Ca^2+^. The rates of rise before and during exposure to HC-056456 were ∼0.03 and ∼0.01 ΔR_SBFI_ min^−1^.

### HC-056456 selectively and reversibly blocks CatSper currents

We have used patch clamp recordings (see Supplemental Fig. S2) to examine the specificity and reversibility of the blockade of CatSper-dependent currents by HC-056456. Applied voltage ramps from −100 mV to +100 mV produced a large inward Na^+^ current that was almost completely blocked by the presence of 2 mM Ca^2+^ (Fig. S2A, inset). This apparent block by Ca^2+^ confounds efforts to record from CatSper from sperm in a physiological medium. However, Fig. S2A shows that smaller CatSper currents could be recorded from cells bathed with solutions containing 700 nM Ca^2+^. The observed current was blocked slightly more than 50% by 20 µM HC-056456 (estimated IC_50_ near 15 µM). In concept, it remains possible that CatSper channel heterogeneity explains residual HC-056456-resistant current.

We also examined the action of HC-056456 on KSper channels, the other major cation channel observed in patch-clamped sperm. The −100 mV to +100 mV voltage ramp produced an outwardly rectifying current that is a mixture of CatSper and KSper in the inward direction, but is primarily KSper in the outward direction (Fig. S2B). As previously observed [19], application of 500 µM quinidine almost completely blocked this current. Subsequent application of 50 µM HC-056456 resulted in partial blockade of this current. For HC-056456 action on KSper we estimate an IC_50_ near 40 µM. Thus HC-056456 is an effective but not perfectly-selective blocker of CatSper channels.

### HC-056456 does not affect HCO_3_^−^ -evoked activation

**Figure 4:**
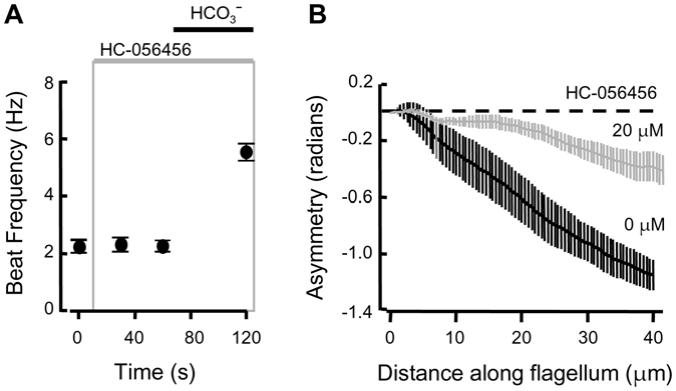
Selective blockade of hyperactivation by HC-056456. (A) Averaged beat frequencies for individual sperm perfused sequentially with medium HS, then HS fortified with 10 µM HC-056456, then with HS containing both 10 µM HC-056456 and 15 mM NaHCO_3_ (N = 7 cells, in 2 experiments). (B) Flagellar waveform asymmetry after pre-incubation under capacitating conditions in the presence (gray) or absence (black) of 20 µM HC-056456. Sperm were transferred back into and perfused with medium HS prior to selection of representative cells for flagellar waveform analysis (N = 13 cells in 3 experiments).

To further assess the specificity of HC-056456 action, we monitored its effects on the HCO_3_^−^-evoked, cAMP-mediated increase of flagellar beat frequency that is known as ‘activation’ [17]. Despite being dependent on external Ca^2+^ [16], HCO_3_^−^-evoked activation does not require the CatSper channel [11], [14]. Fig. 4A shows that for sperm pre-incubated with 10 µM HC-056456, 15 mM NaHCO_3_ still increased beat frequency >2-fold, indicating that HC-056456 has little effect on this major Ca^2+^-dependent, but CatSper-independent, response.

### HC-056456 blocks onset of hyperactivation

The loss-of-function phenotype of CatSper-null sperm includes a lack of hyperactivation of motility [8]–[11], [14], a late step of capacitation that is required for sperm to fertilize zona pellucida-intact oocytes. However, the delayed and asynchronous onset of hyperactivation has obscured whether CatSper channels are required for hyperactivation to initiate, for it to be sustained, or for both. The CatSper channel blocker HC-056456 now allows a pharmacological approach to these questions.

The flagellar waveform analysis developed in our past work measures the time-average of flagellar bending along the proximal flagellum. This asymmetry parameter (compared arbitrarily at 40 µm along the flagellum) increases typically from <0.3 to >1 radian for sperm examined before and after capacitating incubations [11], [14]. Fig. 4B compares asymmetry of wild-type sperm following capacitating incubations in the presence or absence of 20 µM HC-056456 and studied in fresh medium HS. The reduced asymmetry (0.4±0.1 radians) of the sperm treated with HC-056456 indicates a strong blockade of the onset of hyperactivation that is maintained after removal of the drug. Thus prolonged incubation with HC-056456 produces a phenocopy of the lack-of-hyperactivation phenotype of the CatSper null sperm.

### Maintenance of hyperactivation requires open CatSper channels

**Figure 5:**
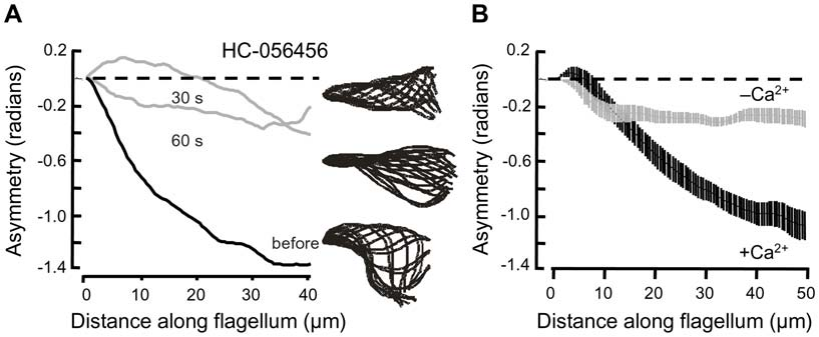
Rapid loss of the flagellar waveform asymmetry of hyperactivation. After incubation under capacitating conditions, sperm were transferred to fresh medium HS. (A) Waveform asymmetry of a representative sperm (analyzed during sequential perfusion with medium HS alone (before, black) and at 30 and 60 s after 20 µM HC-056456 was added to the perfusing medium (gray). On the right, aligned flagellar waveform traces recorded over 2 beat cycles. (B) Averaged waveform asymmetry for sperm bathed in medium HS (+Ca^2+^) or in a Ca^2+^-deficient medium HS (−Ca^2+^) as indicated (7–13 cells in 2 independent experiments).

Using acute application of HC-056456, we now can address the need for CatSper to sustain an established asymmetric beat. Specifically, previously hyperactivated sperm were perfused with medium HS alone, then HS supplemented with 20 µM HC-056456. Fig. 5A shows the waveform asymmetry for a single, representative hyperactivated sperm before and during application of the inhibitor. For this cell, and for 5 other cells examined in trials with 3 other animals, the initially large negative asymmetry (assessed at 40 µm along the flagellum) decreased from ∼1.4 radians before to ∼0.2 radians after 30 and 60 s of exposure to 20 µM HC-056456. These results indicate that functional CatSper channels are required for the maintenance of hyperactivation and that CatSper channels remain open and are not inactivated in hyperactivated sperm.

Some early work found that both the initiation and the maintenance of hyperactivation require external Ca^2+^ [22], but other work reported that hyperactivation continued after removal of external Ca^2+^ [23]. We revisited this question. Waveform asymmetry of individual hyperactivated sperm was examined in HS medium, then in a Ca^2+^-deficient medium HS. Fig. 5B shows that with 2 mM external CaCl_2_, the mean asymmetry at 40 µm along the flagellum was ∼1.2 radian. Within 30–60 s of perfusion with Ca^2+^-deficient medium the asymmetry decreased to ∼0.2 radian, a value similar to that for sperm examined before the onset of hyperactivation [11], [14]. Thus, to maintain hyperactivation, sperm require both open CatSper channels and available external Ca^2+^ to enter through them.

### HC-056456 reversibly removes waveform asymmetry

**Figure 6:**
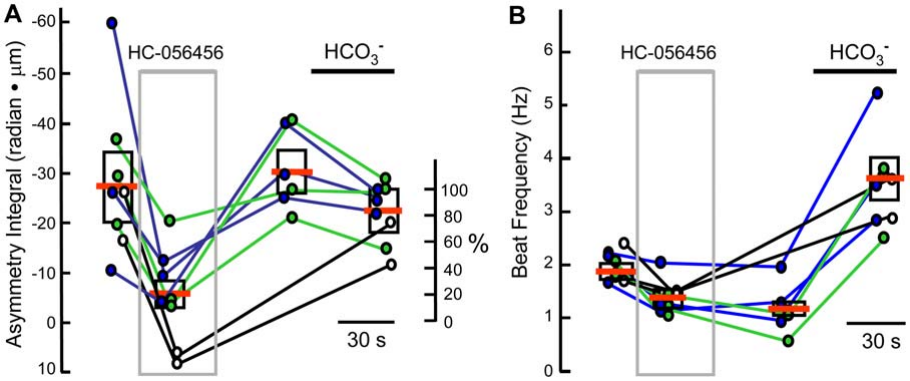
Removal of waveform asymmetry is reversible. After incubation under capacitating conditions, sperm were transferred to fresh medium HS. (A) Waveform asymmetry of individual sperm were examined while sequentially perfusing with HS alone, then at 30 s during a 60 s-perfusion with HS containing 20 µM HC-056456 (gray box), then following 60 s of ‘washout’ with HS alone, and finally following 60 s of additional perfusion with HS containing 15 mM NaHCO_3_. Data for the second and third trials are offset slightly to the right. Boxed bars indicate the mean and SEM of the time-averaged waveform asymmetry integrated over the proximal 50 µm of the flagellum. The right axis normalizes the data to the overall average of the asymmetry integral for the cells during the initial perfusion with HS alone. (B) Flagellar beat frequency from the same image series of the same cells examined in A (8 cells in 3 independent experiments).

In an extension of the experiments of Fig. 5A we asked whether removal of HC-0546456 would allow previously-hyperactivated sperm to recover waveform asymmetry. Fig. 6A shows that (as in Fig. 5A) 30 s of perfusion with HC-056456 decreased asymmetry for each cell examined. The averaged asymmetry integral for the proximal 50 µm of flagellum decreased by >80%. When treatment with the drug continued for another 30 s, several cells (not shown) became immotile and were excluded from analysis. Each surviving cell then recovered an asymmetrical waveform after 60 s perfusion with drug-free medium HS alone, and remained asymmetrical when the HS was fortified with 15 mM NaHCO_3_. These results show that the action of the HC-056456 on waveform asymmetry is quite reversible, but suggests that HC-056456 may have additional actions on hyperactivated sperm. Indeed, Fig. 6B shows that beat frequency decreased during treatment with HC-056456 and remained depressed after 60 s recovery in medium HS. By contrast, Fig. 4A shows that beat frequency was unaltered during 60 s treatment of fresh sperm (examined prior to the onset of hyperactivation). Both fresh (Fig. 4A) and hyperactivated (Fig. 6B) sperm showed substantial acceleration of the flagellar beat when HCO_3_^−^ was applied after removal of HC-056456.

## Discussion

Major considerations in the design of a male-directed, non-hormonal contraceptive include efficacy, safety, reversibility, and ease of delivery [24]. The CatSper channel of sperm has several properties (for review see Navarro et al [25] that make it an attractive target for contraceptive development. First, the required role of CatSper channel proteins in male fertility indicates that a CatSper channel blocker should effectively prevent fertilization. Second, CatSper channel proteins are found only in sperm and spermatogenic cells, indicating that a CatSper-specific blocking drug would have no channel-related actions elsewhere in the body. Third, the CatSper proteins apparently function only in mature sperm, indicating that a CatSper-blocking drug could be delivered only during periods of sexual activity to acutely prevent sperm from reaching and entering the egg. Such actions should be entirely reversible when delivery of drug ceases. By limiting treatment to periods of sexual activity, any cumulative off-target actions would be minimized. Fourth, the CatSper channel proteins in the surface membranes of sperm remain accessible after ejaculated sperm have left the protected environments of the testis and epididymis, indicating that oral delivery of small water-soluble drug could reach CatSper channels through transfer from serum to reproductive fluids. Oral delivery shortly before sexual activity should be a highly-acceptable treatment, with high compliance.

In drug discovery, initial screening of proprietary libraries of ‘*building block*’ compounds reveals active agents (*‘hits’*). With luck, secondary screening of libraries of compounds related to these *hits* reveals something of the required molecular characteristics and identifies a more-limited library to be screened for potency. The most potent of these then become ‘*lead compounds*’ for pharmaceutical development by chemical modification. Large-scale screening efforts require functional expression of the target channel in a cultured cell line [26].

Compound HC-056456 was identified as a *hit* in an initial small-scale screen, and has now been confirmed as a CatSper channel blocker using a new optical method for monitoring of relative CatSper channel activity. This optical approach has the potential for development of a high-throughput method for screening of agents active against the native CatSper channel of intact sperm. Importantly, this method would bypass the barrier imposed by a continued inability to express functional CatSper channels in cultured cells.

Although we learned much about which cellular processes require CatSper from study of the CatSper-null sperm, other approaches were needed to address the temporal aspects of the roles of the CatSper channel. In past work [11] we rescued waveform asymmetry for CatSper-null sperm by application of the local anesthetic procaine. This finding indicates that CatSper has no required role in the prior developmental events that determine the assembly of an asymmetry-competent flagellum during spermiogenesis, or in downstream signaling events that couple Ca^2+^ entry to production of waveform asymmetry in mature sperm.

We have now applied a pharmacological-phenocopy approach to further define the temporal aspects of the required roles of CatSper channel activity. Specifically, we show that acute blockade of CatSper channels with compound HC-056456 prevents the onset of hyperactivation during capacitating incubations, and that application of HC-056456 to already-hyperactivated sperm causes a rapid reversible removal of waveform asymmetry. We can now say with reasonable certainty that open CatSper channels and entry of Ca^2+^ through them are required both to initiate and to maintain hyperactivation.

Unexpectedly, we found that the HC-056456 decreases the beat frequency of hyperactivated sperm, but not of sperm examined prior to capacitating incubations. This suggests either that capacitating incubations make sperm more vulnerable to a hypothetical deleterious action of HC-056456, or that CatSpers have additional roles in controlling the flagellar beat, specifically the beat frequency of hyperactivated sperm. We note that some recent work found that sperm from CatSper-null mice had decreased longevity in vitro [9], [10] and perhaps also in the female reproductive tract [27]. Those findings support the suggestion that additional roles for CatSper channels may be found in future work.

In addition to the pharmacological-phenocopy approach used here for study of the roles of CatSper in activation and hyperactivation of motility, we recently applied a chemical-genetic approach to extend our knowledge of the roles of PKA in the these processes. Using mice whose sperm express a mutant PKA catalytic subunit that is sensitive to the unique cell-permeant inhibitor 1NM-PP1, we found that acute application of the 1NM-PP1 to hyperactivated sperm did not remove waveform asymmetry. Thus, PKA activity apparently is not required to maintain hyperactivation. However, 1NM-PP1 reversibly blocked evoked acceleration of the flagellar beat evoked by HCO_3_^−^, and its application to already-activated mutant sperm produced a rapid return to the slow resting flagellar beat rate [28]. Thus, pharmacological targeting of 2 key components in Ca^2+^-mediated and in cAMP-mediated signaling has advanced our understanding of the temporal requirements of the CatSper channel in maintaining the waveform asymmetry of hyperactivated motility and of PKA in maintaining the accelerated flagellar beat of activated motility.

Further improvements in the available pharmacological tools likely will provide additional insights into the properties, modulation, and roles of the CatSper channel of sperm. Optimization of a high throughput optical assay for native CatSper channel may be a first step in that direction.

## Supporting Information

Figure S1 Chemical structure of compound HC-056456.
(0.03 MB PDF)

Figure S2 HC-056456 selectively blocks CatSper currents in mouse sperm. (A) Current-voltage relationships showing CatSper currents recorded from mouse sperm in response to voltage ramps from −100 to +100 mV. Using the whole-sperm patch-clamp technique CatSper currents were recorded in divalent free buffer (inset) or buffer containing ∼700 nM free Ca^2+^ (trace 1). CatSper currents were blocked ∼55% by the addition of 20 µM HC-056456 (trace 2). Addition of buffer containing 2 mM free Ca^2+^ almost completely blocked the CatSper current (trace 3). Scale bar shows 50 ms on the x axis and 100 pA on the y axis. Inset scale bar is 50 ms on the x axis and 250 pA on the y axis. (B) History plot showing rapid and reversible block of CatSper currents by 20 µM HC-056456 in buffer containing ∼700 nM free Ca^2+^. The HC-056456 decreased currents by >50%, then rapidly returned to the preblock amplitude during washout. Currents were monitored at +80 and −80 mV every 5 s and corrected for the leak currents recorded in medium containing 2 mM Ca^2+^. (C) KSper currents recorded from mouse sperm in response to voltage ramps from −100 to +100 mV. The inward currents observed are a mixture of KSper and CatSper, while the outward currents are composed almost exclusively of KSper (trace 1). Addition of 50 µM HC-056456 blocked slightly more than 50% of the current (trace 2). Addition of 500 µM quinidine almost completely blocked the KSper currents (trace 3). Scale bar is 50 ms on the x axis and 50 pA on the y axis.
(0.02 MB PDF)
