# Supplementary figures and images for: Pharmacological Targeting of Native CatSper Channels Reveals a Required Role in Maintenance of Sperm Hyperactivation
Source: PLoS One. 2009 Aug 31;4(8):e6844. doi: 10.1371/journal.pone.0006844 (PMC2729922; doi:10.1371/journal.pone.0006844)

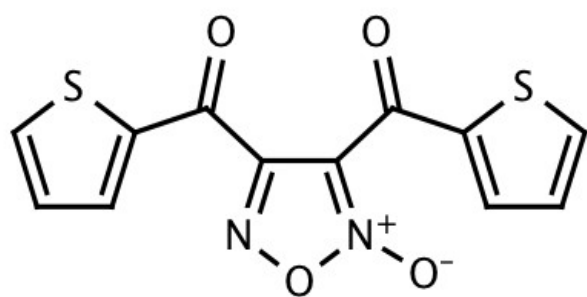

Supplement: Figure S1 — Chemical structure of compound HC-056456. (0.03 MB PDF) [file pone.0006844.s001.pdf]

**A**

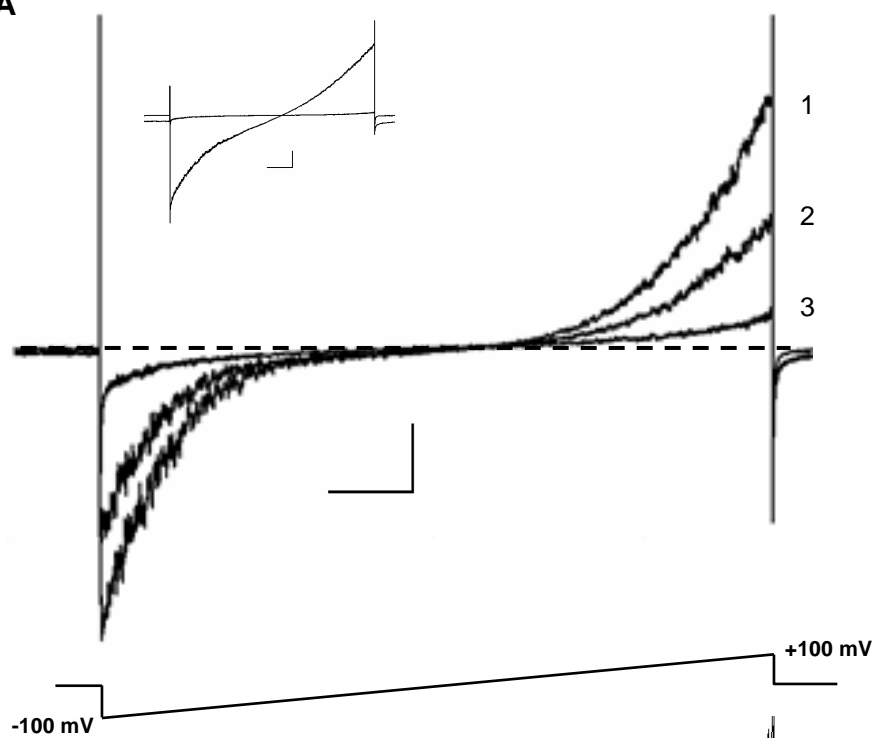

**B**

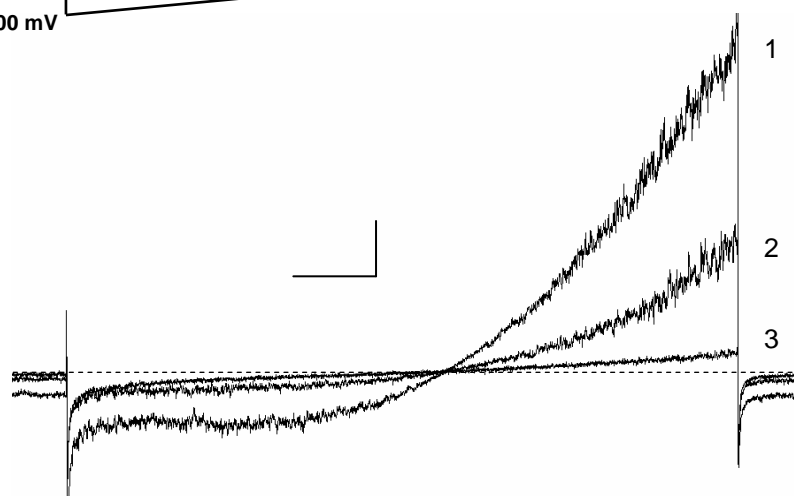

Supplement: Figure S2 — HC-056456 selectively blocks CatSper currents in mouse sperm. (A) Current-voltage relationships showing CatSper currents recorded from mouse sperm in response to voltage ramps from −100 to +100 mV. Using the whole-sperm patch-clamp technique CatSper currents were recorded in divalent free buffer (inset) or buffer containing ∼700 nM free Ca2+ (trace 1). CatSper currents were blocked ∼55% by the addition of 20 µM HC-056456 (trace 2). Addition of buffer containing 2 mM free Ca2+ almost completely blocked the CatSper current (trace 3). Scale bar shows 50 ms on the x axis and 100 pA on the y axis. Inset scale bar is 50 ms on the x axis and 250 pA on the y axis. (B) History plot showing rapid and reversible block of CatSper currents by 20 µM HC-056456 in buffer containing ∼700 nM free Ca2+. The HC-056456 decreased currents by >50%, then rapidly returned to the preblock amplitude during washout. Currents were monitored at +80 and −80 mV every 5 s and corrected for the leak currents recorded in medium containing 2 mM Ca2+. (C) KSper currents recorded from mouse sperm in response to voltage ramps from −100 to +100 mV. The inward currents observed are a mixture of KSper and CatSper, while the outward currents are composed almost exclusively of KSper (trace 1). Addition of 50 µM HC-056456 blocked slightly more than 50% of the current (trace 2). Addition of 500 µM quinidine almost completely blocked the KSper currents (trace 3). Scale bar is 50 ms on the x axis and 50 pA on the y axis. (0.02 MB PDF) [file pone.0006844.s002.pdf]
